# Chitosan Polymer Functionalized-Activated Carbon/Montmorillonite Composite for the Potential Removal of Lead Ions from Wastewater

**DOI:** 10.3390/polym15092188

**Published:** 2023-05-05

**Authors:** Ibrahim Hotan Alsohaimi, Mosaed S. Alhumaimess, Hassan M. A. Hassan, Mohamed Reda, Abdullah M. Aldawsari, Qiao Chen, Mohammed Abdo Kariri

**Affiliations:** 1 Chemistry Department, College of Science, Jouf University, Sakaka 2014, Saudi Arabia; 2 Department of Chemistry, College of Science and Humanities, Prince Sattam Bin Abdulaziz University, Al-kharj 11942, Saudi Arabia; 3 Chemistry Department, School of Life Sciences, Sussex University, Brighton BN1 9QJ, UK

**Keywords:** chitosan polymer, montmorillonite, wastewater treatment

## Abstract

A simple approach for synthesizing a highly adsorbent composite was described for the uptake of heavy metal ions from wastewater. A simple approach for synthesizing a highly adsorbent composite was also described for the elimination of heavy metal ions from contaminated water. The nanocomposite was synthesized via a polymer grafting of chitosan on the activated carbon surface, followed by a stacking process with the layers of montmorillonite clay. The spectroscopic analyses were exploited to confirm the composite structure of the prepared materials. Various adsorption parameters, such as pH, initial concentration, and adsorption time, were assessed. The results showed that the adsorption capacity of the composite for Pb^2+^ ions increased as the pH increased until it reached pH 5.5. The maximum adsorption capacity was observed at an initial Pb^2+^ level of 20 mg/L and a contact time of 150 min. Kinetic models were evaluated, and the pseudo second-order model showed the best match. The adsorption isotherm data were processed by fitting the model with different isotherm behaviors, and the Langmuir isotherm was found to be the most suitable for the system. The maximum adsorption capacity for Pb^2+^ ion on the MMT/CS/AC composite was found to be 50 mg/g at pH 5.5. Furthermore, the composite maintained a high adsorption capability of 85% for five adsorption–desorption cycles. Overall, this composite is envisioned as an addition to the market of wastewater remediation technology due to its chemical structure, which provides influential functional groups for wastewater treatment.

## 1. Introduction

Water treatment is a crucial process that ensures the safety and quality of the water supply. One of the most effective approaches for the uptake of organic compounds from water is adsorption. Adsorption, a cost-effective and environmentally friendly approach for water treatment, is widely used by many municipalities and industries [1,2]. Heavy metal removal from wastewater is receiving considerable focus at the moment because it represents one of the most important environmental challenges worldwide. The adsorption process is widely used for this purpose, and various materials have been efficiently introduced as adsorbents. However, the removal efficiency of the adsorbent depends on many parameters, including adsorbent crystallinity, degree of dispersion, and adsorbent functional groups involved in the chemical structure. Thus, introducing advanced adsorbents with a high removal efficiency of heavy metals is very important.

Clay minerals are paying a great deal of attention as potential adsorbents because they possess a highly ordered layered structure, a high ion exchange property, a low cost, and an increased abundance in nature. Montmorillonite (MMT) is a natural clay with a silicate sheet structure of approximately 1 nm thickness [3]. MMT has potential adsorption properties for heavy metals because of its high cation exchange capacity [4]. Modification studies on the structure of MMT were introduced to increase the MMT surface area and to generate highly porous materials with improved absorptivity [5,6,7]. Exfoliation, grafting, surfactant treatment, and polymer crosslinking were introduced as potential techniques to improve the adsorptive properties of MMT [7,8,9,10,11,12]. Despite all these attempts, further developments and modifications of MMT structure are still needed to adsorb metal ions potentially. Very recently, a composite of MMT with graphene oxide was fabricated as a strong adsorbent for heavy metals. In this composite, a crosslinking agent was used to implement the combination of graphene oxide with the MMT clay. MMT was utilized to boost the dispersibility of GO and also to enhance the graphene oxide absorptivity [13]. Thus, it is interesting to combine different carbon sources in a composite structure with MMT and to investigate the removal behavior of the resultant material.

Activated carbon (AC) is a unique member of the carbon family. The basic structure of AC is approximately the same as the graphite structure. It is comprised of layers of fused hexagons bounded by weak van der Waals forces, and C–C bonds link these layers [14]. AC is one of the most commonly used materials as an adsorbent for heavy metals because it has a unique microporous architecture with an excellent surface area [15]. However, the application of AC as an adsorbent mainly depends on its functionalization process [16,17,18]. Therefore, developing or creating new functional moieties on the AC surface is one of the most important research targets for many researchers. Various techniques using harsh chemicals were developed to functionalize the surface of AC, including acid oxidation, base treatment, impregnation technique, and ozonolysis. Although, these techniques were effective for the surface functionalization of AC, they also sacrificed its unique molecular structure, which is the key property of its high adsorption capacity. Accordingly, searching for environmentally friendly activation techniques of AC is of great significance.

Chitosan (CS) is a biopolymer stemmed from chitin, which is the most abundant natural polymeric material worldwide [19]. Chitosan is known for its excellent adsorption capabilities, making it a popular component of various applications, including water treatment. Its unique chemical structure, which consists of amino and hydroxyl functional groups, provides an excellent surface area and a high capability for the uptake of heavy metals, dyes, and other contaminants.

In the current study, we successfully synthesized an environmentally-friendly multifunctional composite of MMT and AC using chitosan (CS) biopolymer as a crosslinking agent. Specifically, we aimed to offer a highly adsorbent composite with a high surface area by dispersing/recombining the layers of MMT and AC into each other using CS as a binder. The partially protonated amine groups of CS were responsible for their cationic nature [20], allowing it to interact with the polyanionic layers of MMT and AC through electrostatic interaction forces. In addition, the presence of CS is envisioned to boost the uptake efficiency of heavy metals, using the ability of its hydroxyl and amino groups to chelate the heavy metals [21] due to its hydrophilic nature and its structural flexibility. We also discussed the characterization process of the formed composite and showed a comprehensive adsorption study, evaluating the synergetic impact of the nanocomposite on the elimination performance of lead ions from wastewater. Factors such as lead ion concentration, medium pH and temperature, and adsorbent recyclability were examined.

## 2. Materials and Methods

### 2.1. Materials

Chitosan medium molecular weight (CS, 50–190 KDa, degree of deacetylation, DDA, 85%), CH_3_COOH (99%), Pb(NO_3_)_2_ (≥99%), and KOH (≥85%) were provided from Sigma Aldrich, St. Louis, MO, USA. Date palm pits (Phoenix dactylifera seeds) were employed to fabricate activated carbon using the same technique Aldawsari et al. used [22]. Montmorillonite (Cloisite^®^Na; cation exchange capacity 92.6 meq/100 g) was purchased from Southern Clay Products, Gonzales, TX, USA. Deionized water (18.2 MX/cm, produced from Milli-Q, Bay City, MI, USA) was utilized to prepare all the investigated solutions. All the materials employed in the work were employed as received without additional treatments.

### 2.2. Methods

#### 2.2.1. Delamination of AC Layers

A certain quantity of AC was introduced to deionized water. The resultant suspension was submitted to sonication. The sonication process continued for an hour without controlling the medium temperature. After a sedimentation process of 24 h, the portion of the supernatant was separated. The remaining sediments of AC were oven-dried and weighed. The amount of AC in the supernatant portion was estimated from the difference of the starting AC weight and the sedimented AC weight.

#### 2.2.2. Chitosan Polymer/Activated Carbon Composite Formation

CS/AC nanocomposite was prepared as follows. Firstly, a CS polymer solution was fabricated by dispersing 0.1 g of CS in 100 mL of 0.1 M CH_3_COOH. The obtained suspension was agitated overnight to completely disperse the chitosan. Partially protonated chitosan was synthesized by setting the pH at 6.0, utilizing 0.1 M KOH [23]. 1 g of dispersed AC was the introduced to the CS solution. The blend was submitted to sonication for 1 h using a Branson ultrasonic bath, and then stirred overnight at 80 °C to graft CS on the surface of the AC. The obtained composite was separated by a centrifuging process at 10,000 rpm utilizing a digitally operated high-speed centrifuge (Model TGL-16G), and then rinsed many times with aqueous acetic acid (pH 6) to remove unbonded CS moieties. A small portion of the formed composite was vacuum-dried for analysis. 

#### 2.2.3. Composite Formation of Montmorillonite/Polymer Functionalized-Activated Carbon

MMT layers were exfoliated as follows. As a first step, pristine MMT (5 g) was introduced to 100 mL of DI water. The suspension was shaken for 24 h, and then large aggregated and undispersed MMT particles were removed from the dispersion medium by filtration [24,25]. The weight of dispersed MMT remaining in the filtrate was estimated from the difference in weight between the starting and the undispersed. Subsequently, 1 g of dispersed MMT was poured to deionized water, and the final volume was augmented to 100 mL by deionized water. The suspension was then submitted to sonication for 1 h using a Branson ultrasonic bath. At this stage, 1 g of CS/AC composite was introduced to the MMT medium, and the resultant blend was additionally sonicated for 1 h. Finally, the obtained material was separated by centrifugation and vacuum, and labeled MMT/CS/AC [26].

### 2.3. Adsorption Assessment

The uptake of Pb^2+^ ion from an aqueous solution using the prepared composite was conducted in a batch experiment. A 50 mL Pb^2+^ ion (20 mg/L) was typically introduced to 10 mg of composite at a pH of 3–7 at a constant shaking time of 150 min. A series of studies were performed at various periods (5–420 min) at constant Pb^2+^ ions level (20 mg/L), pH (5.5), dose (10 mg), and temperature (25 °C). The Pb^2+^ ion concentration varies from 5–20 mg/L for isotherm study, whereas the other parameters were constant. Utilizing 0.1 mol/L NaOH or 0.1 mol/L HCl, the pH of each solution was adapted to the necessary values. The adsorption efficiency (percentage) and the quantity of Pb^2+^ ion removed were evaluated by Equations (1) and (2):

(1) $$Dye\;Removal,\%=\frac{\left(C_{o}-C_{e}\right)}{C_{o}}\times 100$$

(2) $$Q_{e}({mg\;g}^{-1})=\frac{(C_{o}-C_{e})V_{L}}{w_{g}}$$

where, respectively, C_o_ (mg/L) and C_e_ (mg/L) are the original and the equilibrium concentration of Pb^2+^ ion, m (g) is the sorbent mass of sorbent, and V (L) is the Pb^2+^ ions’ volume.

### 2.4. Desorption Studies

10 mg of the MMT/CS/AC composite was introduced to 25 mL of Pb^2+^ ions solution for the desorption experiment. The resultant mixture was then shaken for 120 min at 100 rpm. The MMT/CS/AC composite that had been covered with Pb^2+^ ions was then filtered. After that, the adsorbent was added to 25 mL of HCl (0.1 M) and agitated continuously for 120 min. The solution and adsorbent were separated, and the adsorbent was then used once more.

### 2.5. Characterization

Fourier-transform infrared spectra (FT-IR) were detected on a Perkin-Elmer FT-IR spectrophotometer. The IR spectra were collected by accumulating sixteen scans at 4 cm^−1^ resolutions. XRD was used to verify the amorphous/crystalline structure of the formed composites. The X-ray spectra were measured on a Shimadzu XRD-7000. Atomic absorption spectrometer (Thermo Scientific, Waltham, MA, USA) was used to determine the concentrations of lead ions. A digitally operated high-speed centrifuge was used to collect the prepared materials.

## 3. Result and Discussion

**Figure 1:**
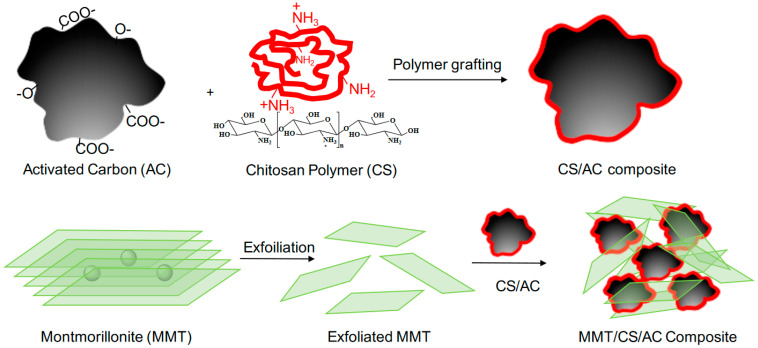
A schematic illustration of MMT/CS/AC composite formation.

Figure 1 Summary of the synthetic route used to synthesize the MMT/CS/AC composite. As seen, AC was grafted with CS polymer through a columbic interaction between the partially protonated amine groups (^+^NH_3_) of CS and the polyanionic functional groups (hydroxyl and carboxyl) of AC [20]. Subsequently, CS functionalized-AC electrostatically interacted with MMT layers, forming the MMT/CS/AC composite.

**Figure 2:**
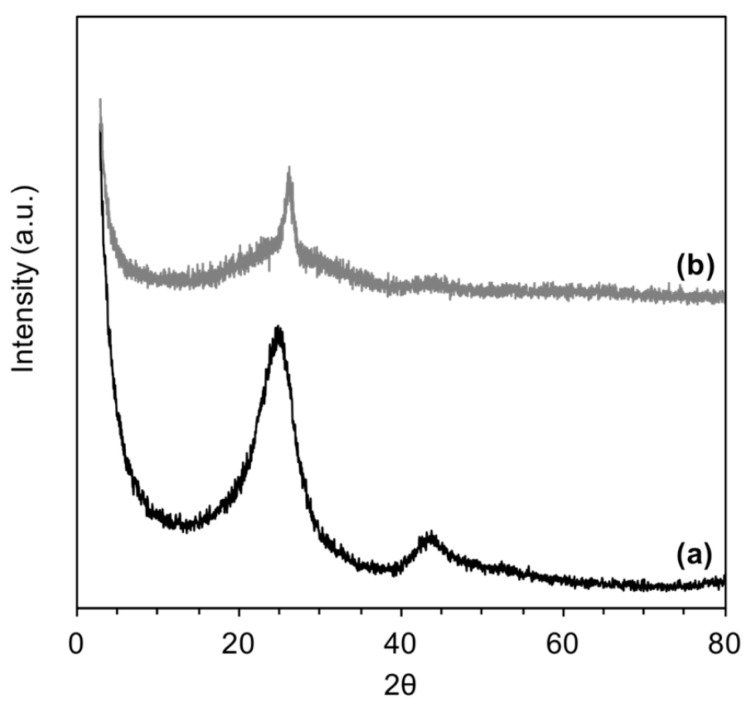
X-ray Diffraction patterns of: (**a**) pristine AC, and (**b**) delaminated AC.

**Figure 3:**
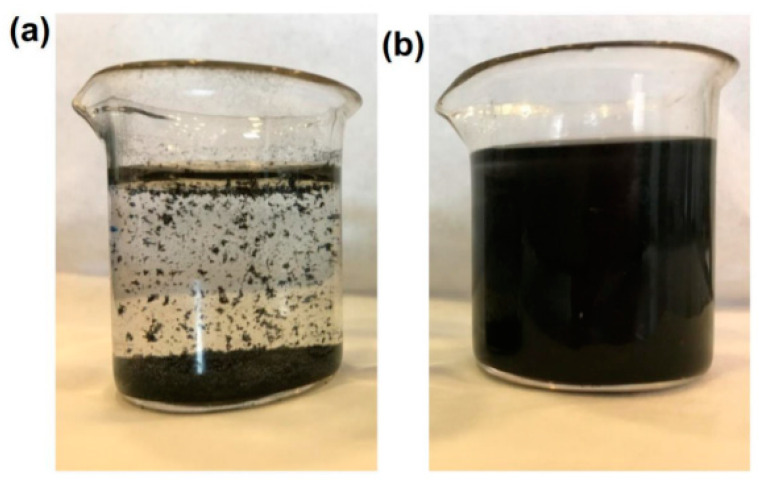
A photo image of AC in deionized water: (**a**) before and (**b**) after grafting with CS.

The amorphous/crystalline structure of AC was confirmed by X-ray analysis. Figure 2 displays the XRD spectra of AC prior to and after delamination. As seen from the spectrum of pristine AC (black spectrum), sharp peaks characteristic of a crystalline structure and aligned layers were observed at 2θ of 25 and 43 [27,28]. As a result of the sonication process of AC, a predominantly amorphous structure was obtained (gray spectrum), suggesting a successful delamination process of the AC layers [29]. This result is beneficial to the elimination behavior of AC. As a result of the grafting of AC with CS, the dispersibility of AC in aqueous media was improved. As seen from the photo images of Figure 3, AC became more attracted to water molecules (capable to create H-bonds) after grafting with CS (Figure 3a). At the same time, the as-received AC showed a fast sedimentation rate (Figure 3b). This result indicated that CS has worked as a dispersing agent, providing AC with other ionic functional moieties on its surface, which is advantageous to the adsorption performance.

**Figure 4:**
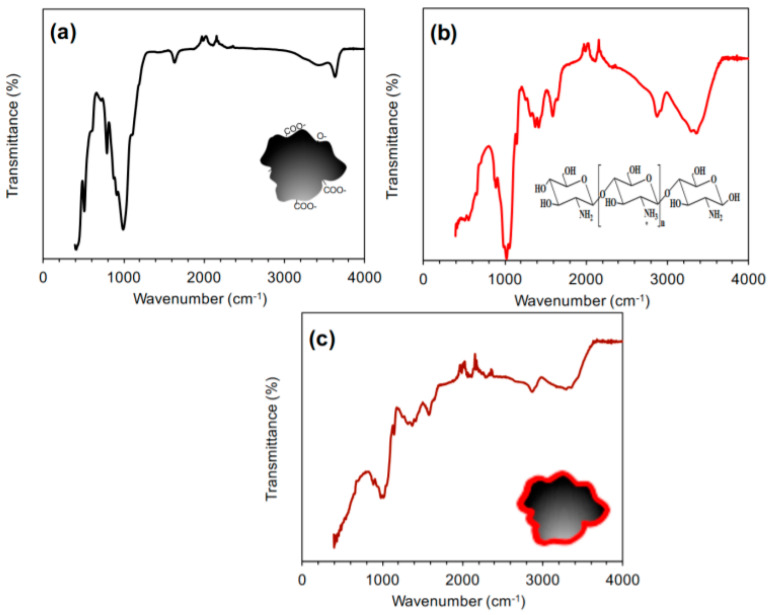
FT-IR spectra of: (**a**) AC, (**b**) CS, and (**c**) CS/AC composite.

The CS/AC composite structure was confirmed by FTIR analysis. Figure 4a shows the FTIR spectrum of AC. The main absorption peaks of AC were noted at 3600, 1650, and 1000 cm^−1^, which correspond to free OH, C=C and C–O groups, respectively [30]. The weak absorption bands recorded at 2100–2000 cm^−1^ were ascribing to bending modes of C–H groups [31].

Figure 4b displays the FTIR spectrum of CS. The absorption bands noted at 3360–3300 and 2930–2860 cm^−1^ relate to the stretching modes of hydrogen-bonded O–H groups that overlapped with the stretching modes of free N–H moieties and the stretching modes of C–H groups, respectively [32,33]. The absorption bands observed at 2100–2000, 1600, 1440–1430, and 1395 cm^−1^ are related to the bending vibration modes of CH, NH_2_ scissoring, CH_2_, and C–O–H groups, respectively. The predominant adsorption bands recorded at 1065–999 cm^−1^ are assigned to the stretching vibration modes of C–N groups overlapped with the stretching modes of the C–O–C group [34,35]. As a result of the grafting of CS on the AC surface, a shift in the absorption band of the N–H group to a lower wavenumber was observed (see Figure 4c). In addition, the band intensity of the free N–H group was decreased and broadened. Moreover, the IR spectrum of the composite showed the absorption bands of both CS and AC. These results suggested a successful interaction between CS and AC. 

**Figure 5:**
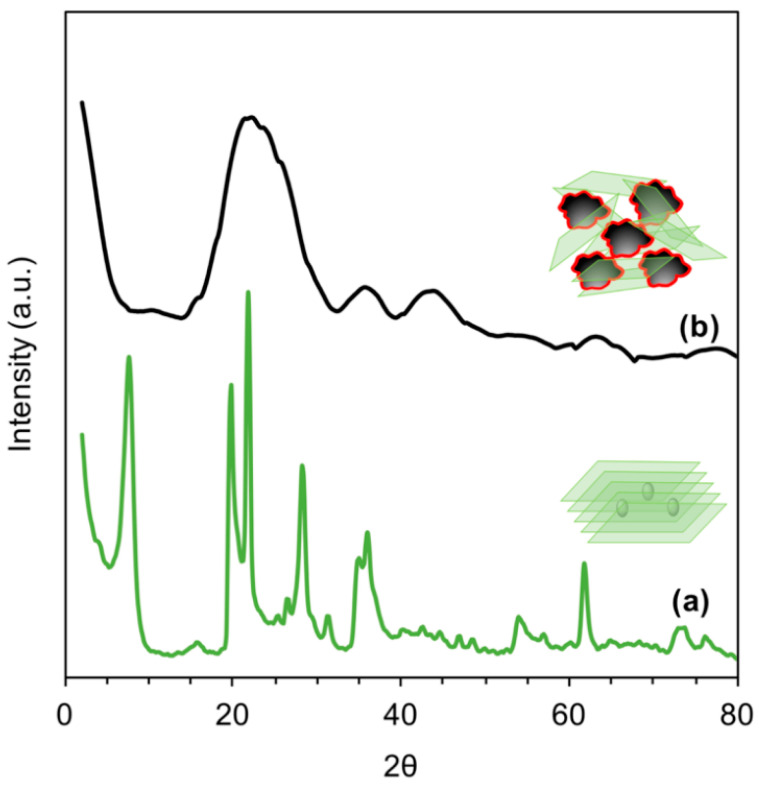
X-ray diffraction patterns of: (**a**) MMT, and (**b**) MMT/CS/AC composite.

The composite structure of MMT/CS-functionalized AC was verified by XRD assessment. Figure 5a shows the X-ray diffraction pattern of MMT. Specifically, the characteristic (001) reflection of MMT was observed at 2θ = 7.4°, from which the basal spacing *d*_001_ calculated by Bragg’s law was 1.2 nm [36]. The peaks recorded at 2θ of 20.0°, 28.2°, 35.0°, 54.1°, and 61.6° are assigned to the typical (100), (005), (110), (210), and (003) reflections of MMT, respectively. The other traces are attributed to the amorphous and poorly crystalline structures of calcites, which are trapped into the MMT composition [37]. Figure 5b shows the X-ray diffraction pattern of MMT interacting with CS-functionalized AC. As seen, the characteristic (001) reflection of MMT almost disappeared, in addition to a decrease in the intensities of the other MMT reflections. A broad diffraction peak at 2θ of 24.5° newly emerged as an indication of the presence of AC moieties. These results suggested a successful exfoliation process of MMT layers and a good restacking with CS-functionalized AC [38].

### 3.1. Batch Adsorption Assessment

#### 3.1.1. Estimate the Optimal Parameters

The impact of different variables such as initial concentration, pH, and uptake time to optimize the removal conditions using the prepared materials was disclosed.

**Effect of pH**

**Figure 6:**
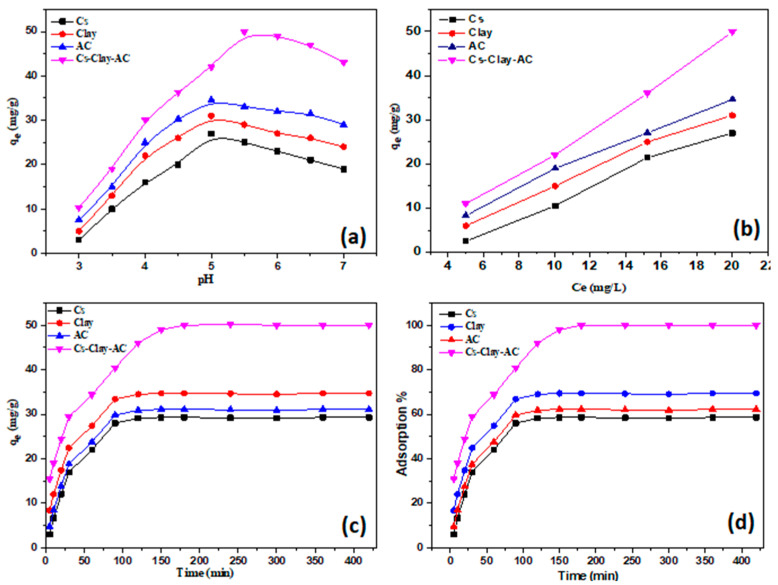
Optimum conditions of the adsorption of Pb^2+^ ions over MMT/CS/AC composite: (**a**) influence of pH, (**b**) influence of initial concentration, and (**c**,**d**) influence of adsorption time of composite.

The solution pH is a vital variable that affects the uptake process. In Figure 6a, the impact of the initial pH value on the uptake of Pb^2+^ ion was shown. It should be noted that Pb^2+^ ion adsorption capacity was attained at pH 5.5. At a low pH, the nanocomposite surface was loaded with a positive charge owing to the protonation of amine moieties. In addition, a significant electrostatic repulsion occurred between the composite surface and the Pb^2+^ ion solution resulting in minimal desorption. However, as the solution’s pH increases, the formation of metal hydroxides leads to a decrease in the efficiency of Pb^2+^ ion removal [22]. The adsorption capacity for CS, MMT, AC, and MMT/CS/AC composite at pH 5.5 was 27, 31, 34.5, and 50 mg/g, respectively. In subsequent studies, pH 5.5 was thus accepted.

**Impact of initial concentration**

The efficacy of Pb^2+^ ion adsorption on the synthesized MMT/CS/AC composite was explored in terms of the initial content of Pb^2+^ ion (5–20 mg/L) at a contact time of 150 min, 10 mg dose, and pH 5.5 (Figure 6b). Pb^2+^ ion removal capability increased from 11–50 mg/g as the concentration of Pb^2+^ rose from 5 to 20 mg/L. This could be linked to the increased adsorption capacity acquired by raising the initial concentration of Pb^2+^ ions due to the accessibility of unoccupied centers.

**Impact of adsorption time**

The influence of adsorption time on the Pb^2+^ ion’s removal capability was evaluated by adsorbent dose (10 mg) at pH 8.5. Obviously, for the fabricated MMT/CS/AC composite, the maximum adsorption capacity and 100% adsorption of Pb^2+^ ion were obtained at an equilibrium time of 150 min (Figure 6c,d). When the contact duration was increased to 400 min, it was evident that the removal of Pb^2+^ ions had not changed much from the measurements made at 150 min, as increasing the contact time resulted in the accumulation of the Pb^2+^ ions, which hinders the diffusion of cumulative ions into the composite. This is the primary reason why there is no significant enhancement in adsorption as compared to that in 150 min.

#### 3.1.2. Kinetics Assessment

**Figure 7:**
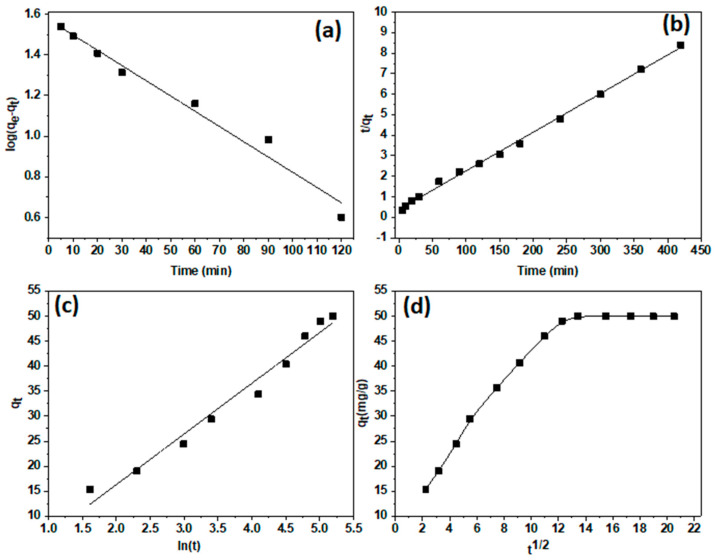
Pseudo first-order (**a**) pseudo second-order (**b**), Elovich (**c**), and intraparticle diffusion model (**d**) for the adsorption of Pb^2+^ ions over MMT/CS/AC composite.

The kinetic model’s evaluation was conducted to highlight the impact of the time of adsorption on the adsorption of Pb^2+^ ions at pH 5.5 on the prepared composite. The pseudo first-order (Equation (3)), pseudo second-order (Equation (4)), and Elovich (Equation (5)) [39] were applied to obtain the relevant kinetics variables:

(3) $$\ln(Q_{e}-Q_{t})=\ln Q_{e}-(\frac{k_{1}t}{2.303})$$

(4) $$\frac{t}{Q_{t}}=\frac{1}{k_{2}Q_{e}^{2}}+\frac{1}{Q_{e}}t$$

(5) $$Q_{t}=\frac{1}{\beta }\ln(\alpha \beta )+\frac{1}{\beta }\ln(t)$$

where the pseudo first-order and pseudo second-order rate constants are k_1_ (min^−1^) and k_2_ (g mg^−1^·min^−1^). Q_e_ and Q_t_ (mg g^−1^) are the same as above. It is possible to calculate the constants α and β from the Temkin plot (Figure 7 and Table 1).

The CS-Clay-AC composite’s pseudo second-order correlation factor (r^2^) was 0.9994, and the calculated adsorption capabilities (Qe) were 48.2 mg/g, in line with the measured ability of 50 mg/g. This information stated that Pb^2+^ ion adsorption on the composite might function well on the pseudo second-order kinetics model (Figure 7b) [40]. The finding were not matched by the pseudo first-order and Elovich kinetics models (Figure 7a,c). In order to gain more understanding of the adsorption mechanism and rate regulatory processes affecting the adsorption kinetics, the kinetic data were additionally coupled to the intraparticle diffusion model. Equation (6) determines the model of intra-particle diffusion:

(6) $$Q_{e}=K_{id}t^{0.5}+C$$

where the intraparticle diffusion rate constants are the k_id_ (mg/g min^−1/2^). The findings disclosed that two separate steps (Figure 7d) tacked the adsorption process. Additionally, the fact that the Q_t_ vs. t^0.5^ plot was unable to pass through the origin suggests that intraparticle diffusion was more than just a rate-control step, and that the border diffusion layer actually had an impact on the removal process [41]. 

#### 3.1.3. Adsorption Isotherms

**Figure 8:**
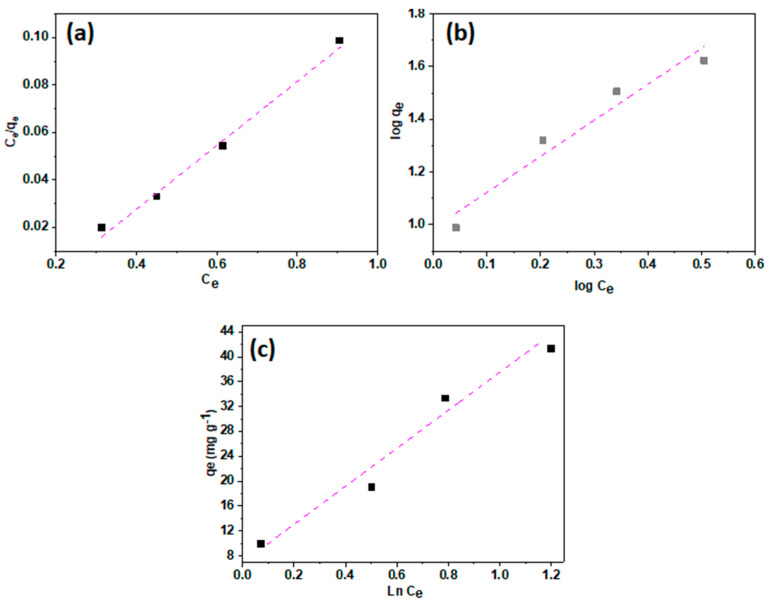
Langmuir isotherm (**a**), Freundlich isotherm (**b**), and Temkin isotherm (**c**) for the adsorption of Pb^2+^ ions on MMT/CS/AC composite.

The isotherms of the adsorption portray how Pb^2+^ ions distribute in the adsorption system at equilibrium between the bulk liquid and the solid state. The processing of the isothermal data by fitting it with distinct isotherm behaviors is a significant factor in exploring the suitable isotherm that can be used for design purposes. The isotherm findings were applied to Langmuir (Equation (7)), Freundlich (Equation (8)), and Temkin (Equation (9)) isotherms:

(7) $$\mathrm{Log}Q_{e}=\log K_{f}+\frac{1}{n}\log C_{e}$$

(8) $$\frac{C_{e}}{Q_{e}}=(\frac{1}{Q_{max}})C_{e}+\frac{1}{Q_{max}K_{L}}$$

(9) $$Q_{e}=B\ln A_{T}+\frac{RT}{b_{T}}lnC_{e}$$

where the amount of Pb^2+^ ion adsorbed at equilibrium (mg/g) and the concentration at equilibrium (mg/L) are Q_e_ and C_e_. K_f_ (mg/g) and n are the Freundlich constants given respectively to the adsorption capacity and intensity. Q_m_ is the highest adsorption amount (mg/g), and k_L_ is the adsorption energy-related Langmuir constant (L/mg). A_T_ is the equilibrium binding constant, (L/g), b_T_ is the adsorption constant (J/mol K), and B is the heat of sorption-related constant (J/mol). The small correlation factor (r^2^) of Freundlich and Temkin isotherms shows that they do not match both models, as shown in Figure 8 and Table 2. In contrast, the synthesized MMT/CS/AC composite’s Pb^2+^ ion adsorption data fit the Langmuir isotherm with the highest regression factor. Finally, the fitting of experimental data allows the ordering of Langmuir, Freundlich, and Temkin by the regression factor (r^2^) of different isotherms. These findings show that the combination of the individual constituents of the composite favors the Pb^2+^ ion’s adsorption in a monolayer coverage [41]. It is, therefore, practical to use MMT/CS/AC composite as adsorbents for the cleaning process with a notable tailored structure.

### 3.2. Reusability Assessment MMT/CS/AC Composite

**Figure 9:**
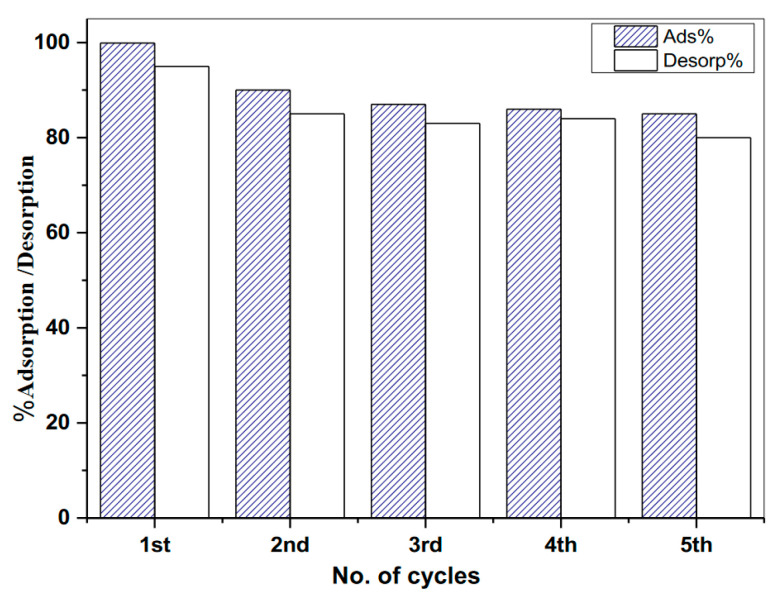
Recycling assessment on MMT/CS/AC composite.

For sophisticated adsorbent materials, recycling, and reusability are significant considerations. According to the pH survey, removing Pb^2+^ ions on the composite was low at smaller pH values, meaning that the removed Pb^2+^ ions can be detached in an acidic medium from the synthesized composite. An acidic solution of HCl (0.1 mol/L) was used in the desorption process because of the petite sizes of the chloride’s ions compared to other counter ions. As shown in Figure 9, a cycling experiment was repeated five times using the same Pb^2+^-loaded MMT/CS/AC composite. The MMT/CS/AC composite maintained a high capacity of adsorption throughout the fifth adsorption-desorption cycle.

### 3.3. Adsorption Mechanism

**Figure 10:**
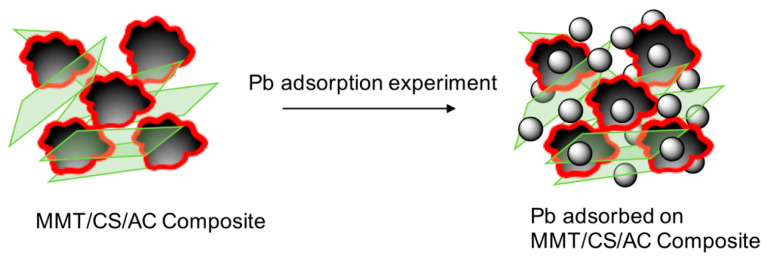
Schematic illustration of Pb adsorption on the formed MMT/CS/AC composite.

The removal process of Pb ions by the synthesized MMT/CS/AC composite was shown in Figure 10. As demonstrated, both the functional moieties of CS (amine moieties), AC (carbonyl moieties), and the negative layers of MMT efficiently contributed to both the capture and the uptake of positive Pb ions from wastewater, showing a high adsorption capability. The removal of Pb ions from contaminated water thus correlated to a chelation process [42]. The removal capacity values obtained in this study exceeded the reported values of various adsorbents for removing Pb(II) ions (Table 3) [43,44,45,46,47,48,49,50,51].

## 4. Conclusions

In conclusion, the simple approach described in this study successfully synthesized a highly adsorbent composite that efficiently removed heavy metal ions from wastewater. A multi-component composite based on CS polymer, AC nanomaterial, and MMT clay was successfully formulated via a grafting/delamination/restacking technique to be a potential composite for the uptake of heavy metal ions from contaminated water. The composite, which consisted of a chitosan-polymer grafting on the activated carbon surface and layers of montmorillonite clay (MMT/CS/AC), exhibited the highest uptake capacity of 50 mg/g for Pb^2+^ ions at pH 5.5. The composite also showed good stability and maintained a high adsorption capability of 85% for five adsorption-desorption cycles. This research highlights the significant of this composite as a potent solution for wastewater treatment and offers a promising addition to the market of wastewater remediation technology.

## Figures and Tables

**Table 1:** The constants of the first-order, second-order, Elovich, and intraparticle diffusion model, and their corresponding adsorption coefficients of Pb^2+^ ion on MMT/CS/AC composite.

Parameters	Adsorbent
	MMT/CS/AC Composite
Pseudo first-order	
r^2^	0.97289
k_1_ (min^−1^)	0.01727
Q_e_ (mg g^−1^)	48.2
Experimental q_e_(mg g^−1^)	50
Pseudo second-order	
r^2^	0.9994
k_2_ (g mg^−1^ min^−1^)	1.056 × 10^−3^
Q_e_ (mg g^−1^)	49.9
Experimental q_e_ (mg g^−1^)	50
Elovich model	
r^2^	0.9706
α	6.437 × 10^−3^
β	9.8 × 10^−4^
Intraparticle diffusion	
r^2^	0.8182
K_int_ (mg g^−1^ min^−1/2^)	1.943
C_i_ (ppm)	18.153

**Table 2:** Adsorption isotherm models and their parameters for Pb^2+^ ions adsorption at 298 K on MMT/CS/AC composite.

q_m,exp._ (mg/g)	Langmuir Isotherm			Freundlich Isotherm			Temkin Isotherm		
	q_m,cal._ (mg/g)	K_L_ (L/mg)	r^2^	K_F_ (mg/g) (L/mg)^1/n^	n	r^2^	b_T_ (J/mol K)	A_T_ (L/g)	r^2^
50	46.3	0.041	0.997	9.71	0.733	0.924	99.7	47.1	0.911

**Table 3:** Comparison of Pb(II) ions removal using various adsorbent.

Adsorbent	Experimental Conditions	Equilibrium Time (min)	q_m_ (mg/g)	Ref.
A commercial activated carbon adsorbent (CGAC)	C_o_: 50–500 mg/L; pH: 7; T: 298 K; m: 2 g	180	20.3	[43]
Citric acid modified pine sawdust (CA-PS)	C_o_: 5–220 mg/L; pH: 5; T: 298 K; m: 0.2 g	120	16.19	[44]
Pb^2+^ adsorption by a compost	C_o_: 2–50 mg/L; pH: 5; T: 298 K; 3.0 g/L adsorbent	1440	21.45	[45]
Biochar-supported graphene oxide composite for removal of lead ion	pH: 5; T: 298 K	900	26.1	[46]
Algal biomass Sargassum glaucescens	C_o_: 0–100 mg/L, 2.0 g/L adsorbent	120	45.8	[47]
Raw agave bagasse	C_o_: 0–100 mg/L, 1.0–2.0 g/L adsorbent	-	35.6	[48]
Coconut tree sawdust	C_o_: 10–200 mg/L, 4.0 g/L adsorbent	90	25	[49]
Manganese oxide coated crushed brick	C_o_: 0–35 mg/L; pH: 5; T: 293 K; 20.0 g/L adsorbent	250	6.42	[50]
MWCNTs/Fe_3_O_4_	C_o_: 5–40 mg/L; pH: 5.3; T: 298 K; 0.5 g/L adsorbent	-	13.04	[51]
MMT/CS/AC	C_o_: 5–20 mg/L; pH: 5.5; T: 298 K	150	50	This study

## Data Availability

Data is contained within the article.
